# 

**Published:** 1871-11

**Authors:** 


### Notice.

There will be a Competitive Examination for resident Physician to the Buffalo General Hospital, on the 1st February, 1872; to date from March 1st, 1872. Applicants are to send their names to Dr. J. N. Brown, Secretary of Staff, No. 115 Franklin Street, when they will be notified of the time and place of examination.

### Books and Pamphlets Received.

Descriptive Catalogue of Medical Works. D. Appleton & Co., 1871.

Remarks on Wines and Alcohol, by Charles A. Lee, M.D.

The prevention of Abscesses in Hypodermic Medication, by Reuben A. Vance, M.D., 1871.

List of the Library Series, commenced by Mr. Henry G. Bohn, now in course of publication by Messrs. Bell & Daldy, London, England.

Clinical Examination of Urine, by Reuben A. Vance, M.D.

The Clinical Thermometer, by Z. C. McElroy, M.D , Zanesville, O.

A contribution to the treatment of the Versions and Flexions of the Unimpregnated Uterus, by Ephraim Cutter, A.M., M.D.

A practical treatise on Fractures and Dislocations, by Frank Hastings Hamilton, A.M., M.D., L.L.D. Fourth edition, revised and improved. Illustrated with three hundred and twenty-two wood cuts. Philadelphia: Henry C. Lea, 1871. Buffalo: Theo. Butler & Son.

Headaches: Their causes and their cure, by Henry G. Wright, M.D. From the fourth London edition. Philadelphia: Lindsay & Blakison, 1871. Buffalo: Theo. Butler & Son.

Restorative Medicine. An Harveian Annual Oration, delivered at the Royal College of Physicians, London, by Thomas King Chambers, M.D., with two sequels. Philadelphia: Henry C. Lee, 1871. Buffalo: Theo. Butler & Son.

The Functions and Disorders of the Reproductive Organs in Childhood, Youth, Adult Age, and advanced Life, considered in their Physiological, Social, and Moral relations, by William Acton, M.R.C.S. Third American edition from the fifth London edition. Philadelphia: Lindsay & Blakiston, 1871. Buffalo: Theo. Butler & Son.

The Druggists General Receipt Book. Comprising a copious Veterinary Formulary; with numerous recipes in patent and proprietary medicines, druggists nostrums, &c.; perfumery and cosmetics; beverages, distetic articles, and condiments; trade chemicals, scientific processes, and an appendix of useful tables, by Henry Beasley. Seventh American, from the last London edition. Philadelphia: Lindsay & Blakiston, 1871. Buffalo: All booksellers.

The Physician's Visiting List for 1872. Philadelphia: Lindsay & Blakiston. Sold by all booksellers and druggists.

On some Disorders of the Nervous System in Childhood. Being the Lumeian Lectures delivered at the Royal College of Physicians of London, by Charles West, M.D. Philadelphia: Henry C. Lea, 1871. Buffalo: Theo. Butler & Son.

The Physician's Dose and Symptom Book, containing the Doses and Uses of all the principal articles of the Materia Medica and Officinal Preparations, &c, by Joseph H. Wythes, A.M., M.D. Tenth edition. Philadelphia: Lindsay & Blakiston, 1871. Buffalo: Breed, Lent & Co.

Emergencies and How to Treat them. The Etiology, Pathology, and treatment of the Accidents, Diseases and cases of Poisoning, which demand prompt action. Designed for Students and Practitioners of Medicine, by Joseph W. Howe, M.D. New York: D. Appleton & Co., 1871. Buffalo: Breed, Lent & Co.

Atlantic Monthly, Little's Living Age, Advance, N. Y. Observer, Peterson's Musical Monthly.

A Text-book of Pathological History: An introduction to the study of Pathological Anatomy, by Dr. Edward Rindflish. Translated from the second German edition with permission of the author, by William C. Kloman, M.D., assisted by F. T. Miles, M.D., with two hundred and eight illustrations. Philadelphia: Lindsay & Blakiston, 1871.

Eating and Drinking; A popular manual of food and diet in health and disease, by George M. Beard, M.D. New York: G. P. Putnam & Sons, 1871.

Hand-Book of Skin Diseases, by Dr. Isidor Neumann. Translated from the second German edition, with notes, by Lucius D. Bulkley, A.M., M.D. Illustrated with sixty.six wood-cuts. New York: D. Appleton & Co., 1872. Buffalo: Breed, Lent & Co.

Containing Original Articles, Reports of Medical Societies and Hospitals, Editorial, Reviews, Correspondence, Army News, etc., etc.; including the usual variety of Medical Periodical Publications. Specimen copies sent on application.

Address, Buffalo Medical & Surgical Journal, Buffalo, N. Y.

TERMS OF SUBSCRIPTION, - - $3.00 PER YEAR, IN ADVANCE.

